# Pre-adult exposure to three heavy metals leads to changes in the head transcriptome of adult flies

**DOI:** 10.17912/micropub.biology.000591

**Published:** 2022-07-02

**Authors:** Kenton R Felmlee, Stuart J Macdonald, Elizabeth R Everman

**Affiliations:** 1 Department of Molecular Biosciences, University of Kansas; 2 Department of Molecular Biosciences, University of Kansas; Center for Computational Biology, University of Kansas

## Abstract

We examined the effect of developmental exposure to three heavy metals - cadmium, copper, and lead - on gene expression in adult head tissue in the model organism *Drosophila melanogaster* . All metals affected development time and/or gene expression level. While variation in the response to each metal was apparent, two differentially-expressed genes were upregulated in response to all three metal treatments, and 11 genes were downregulated in two of the three treatments. Our work reveals that developmental metal exposure has the potential to have long-lasting, metal-specific effects on gene expression in adults, even after the metal stress has been removed.

**Figure 1. Response to heavy metal exposure during development:**
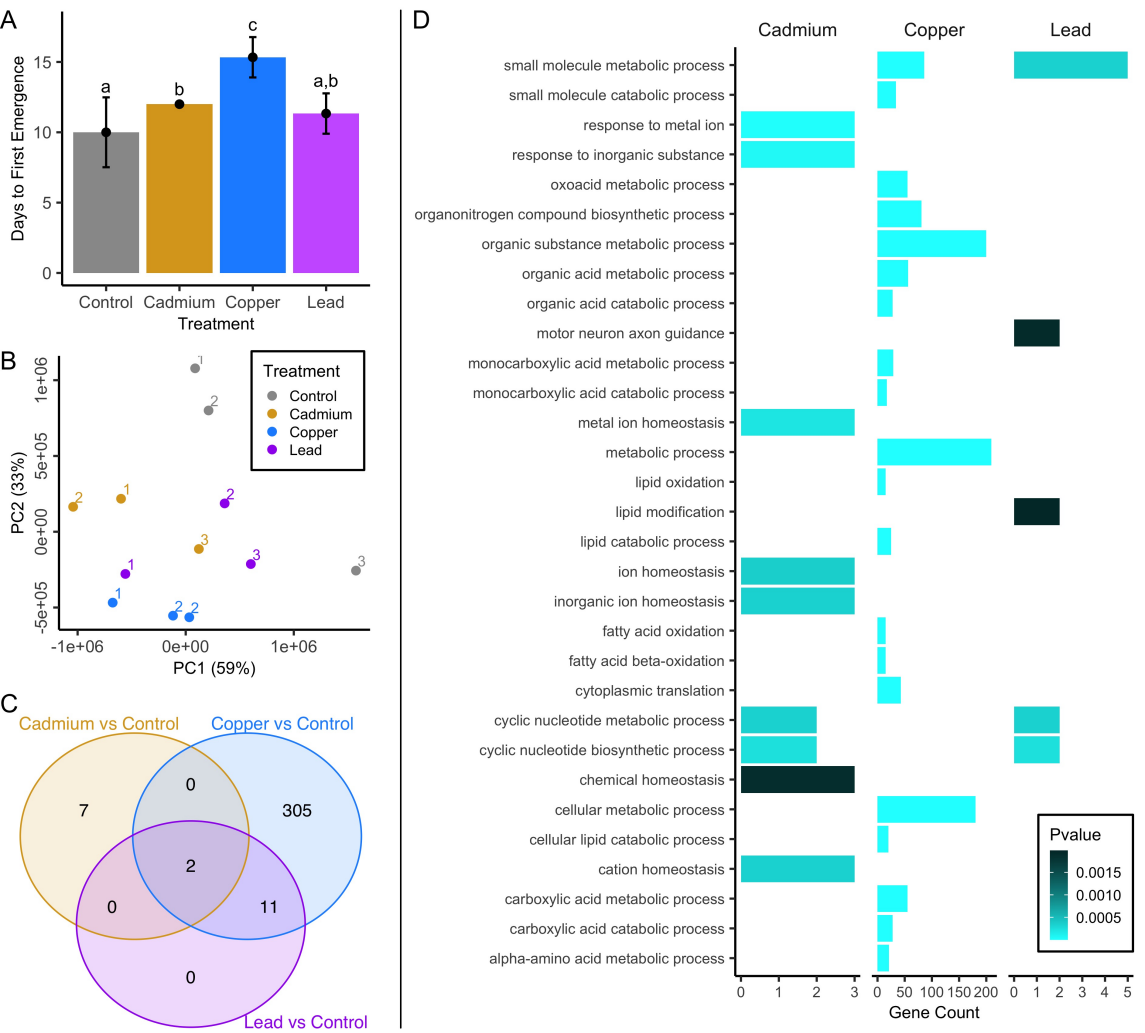
(A) Metal-contaminated rearing media significantly increased development time relative to the control (Treatment: F _3,8_ = 37.1, P < 0.0001). Tukey’s HSD pairwise comparisons revealed that copper-treated embryos had significantly longer development time than all other treatments (adj P < 0.005). Letters above bars represent significant differences at an experiment-wide alpha = 0.05. Points indicate group means and error bars denote 95% CI. (B) Principal components analysis of raw count data for all genes shows clustering of the 12 samples is consistent with treatment. The block from which the sample was derived is indicated above each point. (C) Overlap in DE genes identified for each metal treatment relative to the control. (D) Gene Ontology (GO) enrichment for each set of metal-responsive genes relative to the control condition. The top 20 GO enrichment groups are shown for the copper response.

## Description

Heavy metal pollution presents an important health risk to populations worldwide. Risks are especially high for children who live in communities that are subject to high levels of mining activities, or experience outdated infrastructure, such as lead in paint and water pipes (Blaurock-Busch *et al.* 2011; Stuart and Zeager 2011; Horton *et al.* 2013; Rodríguez-Barranco *et al.* 2014; Knoblauch *et al.* 2017). Exposure to heavy metals during development can cause irreversible damage to neurological function, increase risk of developing ADHD and autism, and can exacerbate susceptibility to blood-related conditions such as anemia (Turgut *et al.* 2007; Woods *et al.* 2014; Hsueh *et al.* 2017; Lee *et al.* 2018). Understanding how exposure to heavy metals during development alters gene expression in adults can provide deeper insight into the effects of heavy metal exposure on cognitive function.

Our goal in this study was to test the effects of cadmium, copper, and lead exposure during development on gene expression in adult flies, and to characterize any similarities in the patterns of differential gene expression observed in the three metal treatments. We used *Drosophila melanogaster* as our model system, given the ease of rearing experimental individuals, the highly conserved metal response pathways between flies and humans (Calap-Quintana *et al.* 2017; Zhou *et al.* 2017), and the active research that leverages this model for understanding the health effects of heavy metal toxicity (e.g. Ruden *et al.* 2009; Zhou *et al.* 2016, 2017; Everman *et al.* 2021).

**Heavy metals delay development:** We exposed embryos from a single inbred wildtype strain to 2mM copper(II) sulfate (CuSO _4_ ), 0.5mM lead acetate (Pb(C _2_ H _3_ O _2_ ) _2_ ), and 0.1mM cadmium chloride (CdCl _2_ ), recorded the time to first emergence of adult flies, and collected adult female heads for mRNA sequencing. We expected exposure to heavy metals to delay development, as this pattern has been reported in *D. melanogaster* for other metals, including copper and lead (Hirsch *et al.* 2009; Everman *et al.* 2021). Indeed, we found developmental heavy metal exposure significantly delayed the time to first emergence by an average of 2.9 days relative to the control (Treatment: F _3,8_ = 37.1, P < 0.0001; Figure 1A). The negative effect of treatment was primarily driven by the copper treatment, which delayed development by an average of 5.3 days relative to the control (Figure 1A, P = 0.00004). Cadmium very slightly increased development time relative to the control (Figure 1A, P = 0.02), while lead had no effect (Figure 1A, P = 0.13). It is likely that the apparently different levels of stress induced by each treatment is due at least in part to the different metal concentrations employed.

**Heavy metal exposure during development alters adult gene expression:** Previous work has focused on examining the regulatory effect of stress directly in the life stage during which the stressor is applied (Yepiskoposyan *et al.* 2006; Everman *et al.* 2021; Frat *et al.* 2021; Yang *et al.* 2022). Our work specifically tests the hypothesis that exposure during the earlier life stages (larvae) will continue to influence gene expression in the following life stage (adults), even after the source of stress has been removed. Here, flies developed in metal-contaminated media, adults were (at most) exposed to the rearing media for 24 hours post eclosion, but test adults were not exposed to metal for at least 2 days prior to tissue harvesting.

Preliminary examination of raw gene expression counts revealed clustering of the 12 samples by treatment, with PC1 accounting for 59% of variation and PC2 accounting for 33% of variation (Figure 1B). Replicates for each treatment were collected across three blocks, except for two copper-exposed samples derived from block 2 (and zero from block 3), which clustered very closely. PC1 generally separates metal-exposed from control samples and PC2 generally separates each of the treatment-specific sample groups (Figure 1B).

Developmental exposure to each metal resulted in differential expression of genes in head tissue of adult females. Relative to the control, we saw 318 differentially expressed (DE) genes for copper, 9 for cadmium, and 13 for lead using a false discovery rate (FDR) threshold of 5%. DE genes influenced by copper and lead were primarily downregulated in response to developmental metal stress (copper = 93.1% downregulated, lead = 84.6% downregulated), while DE genes influenced by cadmium were primarily upregulated (cadmium = 66.7% upregulated). The extended data for each metal includes significantly DE genes at *p* = 0.05 along with mean expression and log2 fold-change.

Because the metal treatments applied in this study resulted in phenotypically-distinct levels of stress, we did not statistically compare metal-specific responses in gene expression. However, we did find some overlap in the gene expression response to each metal treatment (Figure 1C). Notably, two genes were upregulated in all 3 metal treatments: *Gyc76C* (FBgn0266136) and *pre-mod(mdg4)-U* (FBgn0261845). *Gyc76C* is expressed in the nervous system (Liu *et al.* 1995), and is highly expressed in stress-sensing tissues specifically in response to salt and immune stress (Overend *et al.* 2012). No previous work has linked *Gyc76C* to metal response. Limited information is available on the function of *pre-mod(mdg4)-U.* All thirteen DE genes influenced by lead were also influenced by copper (Figure 1C). This larger set includes *Mtpalpha* (FBgn0028479), a gene involved in mitochondrial function, oxidative stress response, and the response to starvation (Kishita *et al.* 2012), and *Nurf-38* (FBgn0016687), which is involved in chromatin remodeling, responds to insulin signaling (Liu *et al.* 2020), and impacts metamorphosis (Badenhorst *et al.* 2005)). In the lead-tolerant fungus *Phanerochaete chrysosporium* , the ortholog of *Nurf-38* increases in expression in response to lead (Yıldırım *et al.* 2011), although this is the opposite pattern from that we observed here *.* Aside from *Gyc76C* and *pre-mod(mdg4)-U* , all other shared genes between copper and lead-treated samples were downregulated in response to metal stress.

We further examined DE gene lists for patterns indicative of a metal-specific response by testing for gene ontology (GO) enrichment. The 9 genes differentially expressed due to cadmium exposure included three upregulated metallothioneins (log2 fold-change (Log2FC): *MtnD* (FBgn0053192) = 8.22, *MtnA* (FBgn0002868) = 2.58, *MtnB* (FBgn0002869) = 5.76; Cd Extended Data), which are involved in the binding and sequestering of excess metal ions, including cadmium (Egli *et al.* 2006b; Calap-Quintana *et al.* 2017). As a result, we found enrichment for GO terms describing the response to metal ions, inorganic substance, and metal ion homeostasis in the cadmium treatment (Figure 1D). Previous work has shown that metallothioneins respond to cadmium stress (Adams *et al.* 2015) and also play a role in the response to copper and lead-induced toxicity in flies and in mice (Qu *et al.* 2002; Egli *et al.* 2006a; Nanda *et al.* 2019; Everman *et al.* 2021). However, we observed no change in expression of any metallothionein genes for either copper- or lead-exposure at an FDR of 0.05. This pattern held true for copper exposure when considering a nominal significance threshold (p < 0.05), but we did find evidence of increased expression of *MtnD* following lead exposure at this relaxed significance threshold (Log2FC = 3.9, p = 0.002, adj p = 0.31). From the 318 genes differentially expressed due to copper exposure we found enrichment for several terms related to lipid metabolism, and various metabolic processes related to carbohydrates and organic substances (Figure 1D). The 13 DE genes due to lead exposure were enriched for those associated with cyclic nucleotide biosynthesis and metabolism, motor neuron axon guidance, and lipid modification (Figure 1D). Collectively, these results may point to metal-specific patterns in the persistence of gene expression response to developmental metal exposure, but we acknowledge that the differences in concentration, levels of toxicity, and power to detect gene expression change may also play a role.

**Conclusions:** Our experiment demonstrated that developmental exposure to metal-contaminated media influences gene expression in adult flies after they have been removed from the stressor for a short period. This observation suggests that the effects of developmental stress may be long-lasting and have the potential to impact adult traits. This has relevance for understanding the role of toxic metal exposure in young children. Heavy metal exposure in humans is especially concerning given the negative impact on early cognitive development. If developmental exposure results in altered gene expression patterns that last into adulthood, this may provide additional insight into longer term health consequences of metal exposure.

We also found that gene expression patterns were specific to each metal, although we acknowledge that the varying developmental delay associated with each metal implies the concentrations of metals do not result in equivalent levels of stress. Cadmium stress resulted in a precise metal stress response with significant upregulation of three of the five metallothionein genes in *D. melanogaster* . We did not see significant upregulation of metallothioneins for the other metals tested here, possibly due to concentrations used. The concentration of lead we used was not stressful enough to elicit a significant change in development time (Figure 1A), so may not have led to an effect on key metal responsive genes that was maintained into adulthood. In contrast, the concentration of copper we used appears to have been quite stressful (Figure 1A), resulting in widespread metabolic dysfunction (Figure 1D). Future directions will include examining the persistence of the gene expression response into later adulthood, the cognitive and behavioral effects of developmental exposure on adult function, and the impact of metal toxicity during development on adult fitness (e.g., lifespan and egg production). Additional follow-up functional work on the roles of the shared DE genes *Gyc76C* , *Nurf-38* , and *Mtpalpha* may provide further insight into the persistent effects of developmental heavy metal stress.

## Methods

**Fly stocks and environment:** We employed one inbred wildtype strain (RIL 22384) from the DSPR ( *Drosophila* Synthetic Population Resource (King *et al.* 2012)) that we have previously used to characterize the effect of copper on developmental viability and adult survival (Everman *et al.* 2021). All experimental individuals were reared at 25°C, 50% humidity on a 12:12hr Light:Dark cycle.

**Developmental metal exposure:** We reared individuals from embryos to adults on 1.8g instant *Drosophila* media hydrated with 8mL of water, 2mM CuSO _4_ , 0.5mM Pb(C _2_ H _3_ O _2_ ) _2_ , or 0.1mM CdCl _2_ . Informed by previous work, we chose metal concentrations that were expected to result in some mortality and/or developmental delay, but were not expected to lead to high levels of mortality (Maroni and Watson 1985; Zhou *et al.* 2016; Everman *et al.* 2021). To obtain embryos, mixed sex adults from DSPR strain 22384 were placed on cornmeal-molasses-yeast media overnight, and embryos were collected using a mounted needle and transferred to vials containing metal-contaminated or control media. The experiment was carried out in three blocks, each consisting of three replicate vials per treatment, with 50 embryos per vial (except for block 3 where we included five replicate vials of the copper treatment).

Once adults began emerging, flies were moved within 24 hours of eclosion to new cornmeal-yeast-molasses media and held for a period of 2-7 days (average 4 days). Within each block and treatment, flies emerging from the 3 replicate vials were mixed and aged. Females were then harvested into groups of 10 via CO _2_ anesthesia one day prior to flash freezing in liquid nitrogen, and stored at -80°C. Because very few flies emerged from the copper treatment from block 3, we obtained the third replicate of copper-treated females for sequencing from block 2.

**Sample preparation and sequencing:** Heads were isolated from females by briefly vortexing frozen flies (3-5 seconds) and manually sorting heads from bodies on the surface of an aluminum dry bath block chilled with dry ice to prevent tissue thawing. Head tissue was stored at -80°C prior to RNA extraction.

To extract RNA, we added 300uL TRIzol and 3-6 2.7mm glass lysis beads to each sample of ~10 heads and homogenized tissue using a BioSpec Mini-BeadBeater-96 for 45 seconds. RNA was extracted using the Direct-zol RNA microprep kit following the manufacturer’s instructions. mRNA sequencing libraries were made for each sample, starting with 200ng total RNA, using the Illumina stranded mRNA Prep ligation-based kit with unique sample-specific indexes. All 12 libraries were quantified using the Qubit dsDNA BR kit, pooled based on concentration, and a 1X AMPure XP bead cleanup was performed on the pool to remove a small fraction of adapter dimers prior to sequencing. The pooled sample was sequenced at the KU Genome Sequencing Core on an Illumina NextSeq2000 P2 Flow Cell, generating ~36.7M 50bp paired-end reads per sample. RNAseq reads are available from NCBI SRA BioProject PRJNA843717.

**Data processing and analysis:** After confirming that the developmental timing data met assumptions of parametric tests (Shapiro Wilk’s Test for normality: w = 0.9, P = 0.2, Levene’s Test for Homogeneity of Variance: F = 0.9, P = 0.5), the effect of metal exposure on development time was analyzed using a one-way ANOVA, testing the effect of treatment (four levels) on the number of days between embryo collection and the emergence of the first adult fly. We used Tukey’s HSD to test all pairwise comparisons of the four treatment levels and performed all analyses in R (v. 4.1.3) (R Core Team 2017).

We used fastp (v. 0.23.2) (Chen *et al.* 2018) to assess sequence read quality and to trim bases with mean quality scores less than 30 using a window size of 5bp. PE reads were aligned and quantified using salmon (v. 1.8.0) (Patro *et al.* 2017), which employs a computationally-efficient quasi-mapping strategy and aligns reads to a transcriptome (FlyBase transcriptome release 6.44). We used principal components analysis to assess sample clustering and look for outliers using the prcomp function in R. Differential expression (DE) analysis was performed with DESeq2 (v. 1.34.0) (Love *et al.* 2014) in R, fitting the model ( ~ Treatment), where Treatment includes four levels: copper, lead, cadmium, and water control. The control treatment was set as the reference level and differential expression was assessed for each metal-treated sample relative to the control. Significance was determined using Benjamini-Hochberg multiple test correction for false discovery rate with a threshold of 5% FDR. Because each of the metal concentrations resulted in different levels of toxicity, we did not statistically compare gene expression among the metal treatments.

To examine DE gene lists for evidence of gene ontology enrichment, we used the Bioconductor annotation package org.Dm.eg.db (v. 3.8.2) (Carlson 2019) and the GO analysis R package GOstats (v. 2.60.0) (Falcon and Gentleman 2007).

## Reagents

**Table:** 

**Fly Rearing**	**Source**
Instant Drosophila Media	Carolina Biological Supply Company 173200
Copper(II) Sulfate	Sigma Aldrich C1297
Cadmium Chloride	Sigma Aldrich 655198
Lead(II) Acetate Trihydrate	Sigma Aldrich 32307
	
**Drosophila Strains**	**Source**
22384	Drosophila Synthetic Population Resource; Macdonald Lab; (King *et al.* 2012)
	
**Sample Preparation**	**Source**
TRIzol Reagent	ThermoFisher 15596018
Illumina stranded mRNA Prep, Ligation kit	Illumina 20040534
IDT for Illumina RNA UD Indexes Set A, Ligation (96 Indexes, 96 Samples)	Illumina 20040553
Direct-zol RNA Microprep	Zymo R2060
AMPure XP beads	Beckman A63881

## Extended Data

Description: Cd Extended Data. Resource Type: Dataset. DOI: 10.22002/D1.20208

Description: Cu Extended Data. Resource Type: Dataset. DOI: 10.22002/D1.20209

Description: Pb Extended Data. Resource Type: Dataset. DOI: 10.22002/D1.20210
